# The aphrodisiac potential of β-cyclodextrin–curcumin via stimulating cAMP-PKA pathway in testicular Leydig cells

**DOI:** 10.1038/s41598-022-18065-3

**Published:** 2022-08-22

**Authors:** Liu Yang, Shan Xue, Lin Yuan, Zihan Li, Haitao Hu, Yichang Zhang, Yimei Liu, Juan Li

**Affiliations:** 1 Department of Pharmacy, Wuhan Hospital of Traditional Chinese Medicine, Wuhan, 430014 Hubei China; 2 Hubei Province Key Laboratory of Traditional Chinese Medicine Resource and Chemistry, Department of Pharmacy, Hubei University of Chinese Medicine, Huang-Jia-Hu West Road 16#, Hongshan district, Wuhan, 430065 Hubei China; 3 Hubei Provincial Key Laboratory of Occurrence and Intervention of Rheumatic Diseases, Hubei Minzu University, Enshi, 445000 Hubei China

**Keywords:** Molecular biology, Health care

## Abstract

The water-soluble β-cyclodextrin–curcumin (CDC) is used in pharmaceutical applications and as a natural food colorant. The previous study revealed that curcumin potentially impacted the reproductive system. The present study investigated the possible roles of the CDC in testosterone secretion in Leydig cells and mice. Primary Leydig cells were treated with the CDC to determine their effect on cell proliferation, testosterone levels, the protein and mRNA expression of the transcription factor, and steroidogenic enzymes. Our data showed that CDC stimulated testosterone production via upregulating transcription factor steroidogenic factor-1 (NR5A1), cAMP-response element-binding protein (CREB), and steroidogenic enzymes steroidogenic acute regulatory protein (StAR), cholesterol side-chain cleavage enzyme (CYP11A1), 17-alpha-hydroxylase/17,20-lyase (CYP17A1), 3β-/17β-hydroxysteroid dehydrogenase type 1 (3β/17β-HSD, HSD3b1/HSD17b1). CDC could significantly stimulate H89-suppressed StAR and CREB expression but not reverse melatonin-suppressed StAR expression. We further detected the hormonal activity with transgenic yeast, and CDC showed potential androgenic antagonistic activity. Meanwhile, we investigated its aphrodisiac effect on hydrocortisone-induced mice. Exposure to hydrocortisone decreased the mating ability, reproductive organs, and testosterone level and disrupted testicular histology. However, all of these effects were significantly improved by CDC treatment. In conclusion, these results indicated that mechanisms of CDC in stimulating testosterone production involve upregulating the cAMP-PKA pathway.

## Introduction

Curcumin, a diferuloylmethane, is naturally present in turmeric (*Curcuma longa*). It’s added as a spice or natural colorant and is considered an herbal remedy. Recent studies have confirmed the potential pharmacological actions of curcumin in inflammatory disorders, metabolic syndrome, cardiovascular disease, and neurological disorders^1^. Beyond these beneficial properties, recent studies also revealed that curcumin potentially impacts the reproductive system. It’s reported that dietary curcumin supplementation improved testis histological parameters in aged broiler breeder roosters^2^. Besides, curcumin was found to have curative potential on the reproductive system function and its impairment, regulated by stress and reproductive-related hormones^3^. Of note, researchers also demonstrated that curcumin could increase spermatozoon motility in metronidazole-treated mice^4^.

However, the clinical application of curcumin is considerably limited due to its instability, low aqueous solubility, and poor bioavailability^5^. The approaches to increasing the bioavailability of curcumin include the use of nanoparticles, liposomes, micelles, and phospholipid complexes^6^. Cyclodextrins have been considered attractive candidates for nano-drug delivery systems due to their commercial availability, easy functionalization, low immunogenicity, biocompatibility, and safety^7–9^. The use of cyclodextrins led to significant increases in solubility and bioavailability of steroid drugs, such as testosterone and progesterone, and improvement in the efficacy and safety of these drugs^10^. β-Cyclodextrin derivatives can solubilize steroids and improve the bioavailability of these hydrophobic steroidal drugs^11^.

Recently, the water-soluble curcumin complex has been a commercially available nutritional supplement. The CDC used in this search is developed by a β-CD mediated curcumin drug delivery system via encapsulation^12^. Reddy et al.^13^ reported that the curcumin-C3 CDs showed an increased entrapment efficiency of 97.8% and improved antioxidant activity compared to curcumin. Also, the curcumin-CD complex showed superior water solubility (3.72–70 μg/mL)^14^, in vitro release performance, and higher cytotoxicity^15^. Thus, this study will evaluate the possible aphrodisiac potential of CDC in Leydig cells, yeast cells, and mice.

Leydig cells of the testes are responsible for the biosynthesis and secretion of testicular androgen testosterone in the male, and testosterone is essential for spermatogenesis. Cholesterol is the precursor for testosterone synthesis and is transported to mitochondria by StAR, which serves as the rate-limiting step in steroidogenesis^16^. The translocated cholesterol is then converted to pregnenolone by P450scc (P450 side-chain cleavage, CYP11A). Subsequently, pregnenolone is converted to testosterone in the steroidogenic cascade, regulated by steroidogenic enzymes, such as CYP17A1 and 3β/17β-HSD^17^. Meanwhile, NR5A1 and pCREB act as transcription factors to regulate CYP11, HSD3B1, and StAR expression. Therefore, this study aimed to investigate the possible aphrodisiac mechanism of the CDC in the cAMP/PKA signaling pathway, including transcription factors (NR5A1 and CREB) and steroidogenic enzymes (StAR, CYP11A1, CYP17A1 and 3β/17β-HSD).

## Materials and methods

### Sample preparation

Curcumin (Batch No. 0823-9802) was purchased from the National Institute for the Control of Pharmaceutical and Biological Products. CDC prepared by the co-precipitation technique was collected from Hubei Tianji Chinese Medicine Pieces Co., Ltd. in China. It is a bright yellow solid and easily soluble in water without precipitation and floating particles. Reversed-phase high-performance liquid chromatography (HPLC) was performed on a Dionex HPLC system with a P680 Pump, an Agilent ZORBAX SB C18 column (4.6 mm × 150 mm, 5 μm), and a UVD 170U UV–Vis variable wavelength detector. The detailed method was according to the *Chinese Pharmacopoeia* 2015. The content of curcumin was 20%.

### Animals

Kunming mice (18–22 g) and male Sprague–Dawley rats (180–220 g) were purchased from the Hubei Provincial Center for Disease Control and Prevention (SCXK 2015-0018; Wuhan, China). Animal experiments were approved by the Institutional Animal Care and Use Committee and the local experimental Ethics Committee (Laboratory Animal Certificate no. SYXK2017-0067). All methods were carried out following relevant guidelines and regulations, and this study was carried out in compliance with the ARRIVE guidelines.

All animals were maintained under a controlled temperature of 24 ± 2 °C and humidity of 55 ± 15%, with access to food and water ad libitum and a 12-h day/night cycle.

### Cell culture and treatment

Leydig cells were isolated from 50 to 70-day-old Sprague Dawley rats by the combination of enzyme digestion and Percoll separation, as previously described^18,19^ with some modifications. In brief, decapsulated testes were placed into an enzymatic solution containing 0.05 mg/mL collagenase I (Invitrogen) for 15 min at 34 °C with gentle shaking. After incubation, the digestion was stopped by the DMEM-F12 culture medium containing 9% fetal bovine serum, 1% horse serum, 1% 0.5 mM sodium pyruvate, and 1% penicillin–streptomycin, and the solution was filtered through a 70-μm nylon strainer.

The low levels of horse serum helped maintain Leydig cell survival and steroidogenic function, and 10–20% fetal bovine serum promoted the attachment of cultured Leydig cells to the culture substratum^20^. Meanwhile, pyruvate was superior to glucose as an energy source to support steroidogenesis^21^. Then, the dispersed cells were washed with DMEM/F12 and layered over a Percoll gradient (5%, 30%, 58%, and 70%; Biosharp, Wuhan, China). The gradient was centrifuged for 35 min at 800*g*, and cells localized between Percoll gradient 70 and 58% were isolated (the second layer). After the repeating wash steps of the medium, the Leydig cells were incubated in the DMEM-F12 culture medium. Cell viability determined by the trypan blue test was more than 90%.

The purity of Leydig cells was determined by 3β-HSD histochemical staining^22^. Leydig cells were incubated in the 24-well plates with a 0.4 mL/well 3β-HSD staining solution. The staining solution contained 0.01 M PBS supplemented with 0.1 mg/mL nitro-blue tetrazolium (Biosharp, Wuhan, China), 1.0 mg/mL nicotinamide adenine dinucleotide (Shanghai McLean), 0.1 mg/mL dehydroepiandrosterone (Shanghai McLean) and 0.1 mg/mL niacinamide for 90 min at 34 °C. The positive cells were stained a dark blue, and the purity of the Leydig cells was over 90%.

Purified Leydig cells (3 × 10^4^/mL) were plated into 24-well plates and cultured at 34 °C in a humidified atmosphere containing 5% CO_2_-95% air. Two-day cultured Leydig cells grown in the DMEM/F12 culture medium were washed three times in PBS. Then the cells were cultured in a serum-free medium containing different doses of curcumin or CDC for 24 h. 1 IU/mL of human chorionic gonadotrophin (hCG) was used as a positive control. The cytotoxicity assay of control and treated cells was tested by MTT assay by the reported method^23^.

### Measurement of testosterone production

The testosterone concentration in the cell-free culture medium was measured using ELISA assays according to the manufacturer’s protocol (Nanjing Jiancheng Bioengineering Institute, Nanjing, China).

### RT-qPCR (real-time reverse transcription-polymerase chain reaction)

The mRNA expression levels of the *Star, Nr5a1, Cyp11a1, Cyp17a1, Hsd3b1,* and *Hsd17b1* in Leydig cells were analyzed using RT-qPCR analysis. The total RNA of different treatment groups was extracted using Trizol reagent (Invitrogen, Carlsbad, CA, USA), and their quantity and purity were measured by an ultra microspectrophotometer (Thermo Fisher Scientific, USA) based on the absorbance measurement at 260 and 280 nm. The purified RNA was reverse transcribed using a FastQuant RT kit (Tiangen Biotech, Beijing, China) according to the manufacturer’s instructions. RT-qPCR was performed in a LightCycler 480 instrument (Roche, Basel, Switzerland) using the TIANGEN SuperReal PreMix Plus kits (Tiangen Biotech, Beijing, China) with specific primers (Table 1). PCR amplification was initiated by 15 min of denaturation at 95 °C, and then followed by 45 cycles of 95 °C for 10 s, 60 °C for 40 s and 72 °C for 32 s, and a final incubation at 75 °C for 5 min. Analyses were performed using the 2‑ΔΔCq method^24^, and *Gapdh* was used as an internal control.

**Table 1:** The primer sequence.

Primer	Forward sequence (5′–3′)	Reverse sequence (5′–3′)
*Gapdh*	GGCTCTCTGCTCCTCCCTGT	CGTTCACACCGACCTTCACC
*Star*	GGAACCCAAATGTCAAGGAAATCA	CAGGCATCTCCCCAAAGTGTG
*Cyp11a1*	GTCCAGTTGGTCCCACTCCTC	AAGCACCAGGTCGTTCACAATATAC
*Hsd3b1*	GTACATTTATGGGGAGAGAAGTCC	CCAGGCCACATTGCCTACATA
*Hsd17b1*	GGTGGTGCTGCTGTAGAAGAT	TGTTTCAACCCCAATGACTAAG
*Cyp17a1*	GCTCCGAAGGGCAAGTAA	TCCGAGAAGTGCTGCGTAT
*Nr5a1*	TCTCTAACCGCACCATCAAG	TCGACAATGGAGATAAAGGTC

### Western blotting analysis

The protein expression of steroidogenic enzymes and CREB in Leydig cells was detected by western blotting. Leydig cells were separated into 5 groups: the untreated group (Control), the positive group (hCG), the CDC group (0.2, 1.0, 5.0 μM), and H89 (10 μM) or melatonin (10 μM) was also used where appropriate. Proteins were separated by 12% SDS–polyacrylamide gels and were then transferred onto polyvinylidene fluoride membranes. The blots were incubated at 4 °C overnight with specific primary antibodies: against HSD3B1 (A8035), StAR (A16432), NR5A1(A1657), CREB(A11989), and GAPDH (GB11002), all obtained from ABclonal company (Wuhan, China) and diluted 1:1000 in Tris-buffered saline. Subsequently, the membranes were incubated with secondary antibodies (1:2000, HRP-conjugated anti-rabbit IgG, GB23303) for 1 h at room temperature. The membranes were washed with TBS and then visualized using Pierce ECL Western Blotting Substrate (Thermo).

### Yeast estrogen screen (YES) and yeast androgen screen (YAS) assay

The XenoScreen YES/YAS test was performed on 96-well plates to determine hormonally active substances^25–27^. The oestrogenic, anti-oestrogenic, androgenic, and anti-androgenic activities were measured with the XenoScreen XL YES/YAS Assay kit according to the manufacturer’s protocol (Xenometrix, XenoScreen XL YES/YAS, Instructions for Use Version 3.04). Growing yeast cells (*Saccharomyces cerevisiae*) were stably transformed with either hER or hAR and a β-galactosidase reporter system and were exposed to different concentrations of compounds. 17β-Estradiol (E2) and 4-hydroxytamoxifen (4-HT) standards were used as the estrogen agonist and antagonist controls, respectively. For androgen activity, 5α-dihydrotestosterone (DHT) was used as agonist control and flutamide (FL) as antagonist control. Antagonistic activities were measured by evaluating the β-galactosidase signal reduction in yeast cells in 0.2 nM E2 (YES) or 1.0 nM DHT (YAS) in the test medium.

### Evaluation of male sexual behavior

Male mice were randomly divided into 6 groups (*n* = 8). Blank control group: only normal saline as a vehicle. Model control group: 25 mg/kg hydrocortisone intraperitoneally administrated for 7 d from the 4th day of administration^25^. JKSQP group (Jinkui Shenqi Pill from Beijing Tongrentang Company): the same treatment as the model control group and orally administrated with 1.3 g/kg JKSQP. CDC treated group: not only received the same treatment as the model control group but also orally administrated with 1.3 g/kg JKSQP, 50 mg/kg, 150 mg/kg, or 450 mg/kg CDC (about 2, 6, and 18 times to clinical dose). Female mice were brought into estrous by administering estradiol valerate (0.4 mg/kg) 48 h and 6 h before the copulatory study. After 30 daily doses, the female was then introduced into the chamber, and the following sexual behavior parameters were recorded: mount frequency (MF), the number of mounts without intromission from the introduction of the female to ejaculation; intromission frequency (IF): the number of intromissions from the introduction to ejaculation; mount latency (ML): the interval between the introduction and the first mount by the male; intromission latency (IL): the interval from the introduction to the first intromission by the male^28^. Under ethyl ether anesthesia, blood samples were collected from the orbital plexus, and the major organs were then collected. Meanwhile, the testes were fixed in 10% neutral buffered formalin, and the slide sections were stained with hematoxylin and eosin (HE) for light microscopic examination. The testosterone concentration in the mouse serum was detected using the ELISA kits.

### Statistical analysis

Data were presented as the mean ± standard error of the mean. Differences between means were assessed using the one-way analysis of variance followed by Dunnett’s *t*-test with GraphPad Prism 8. *P* < 0.05 or *P* < 0.01 were considered to indicate a statistically significant difference.

## Results

### Leydig cell viability

**Figure 1:**
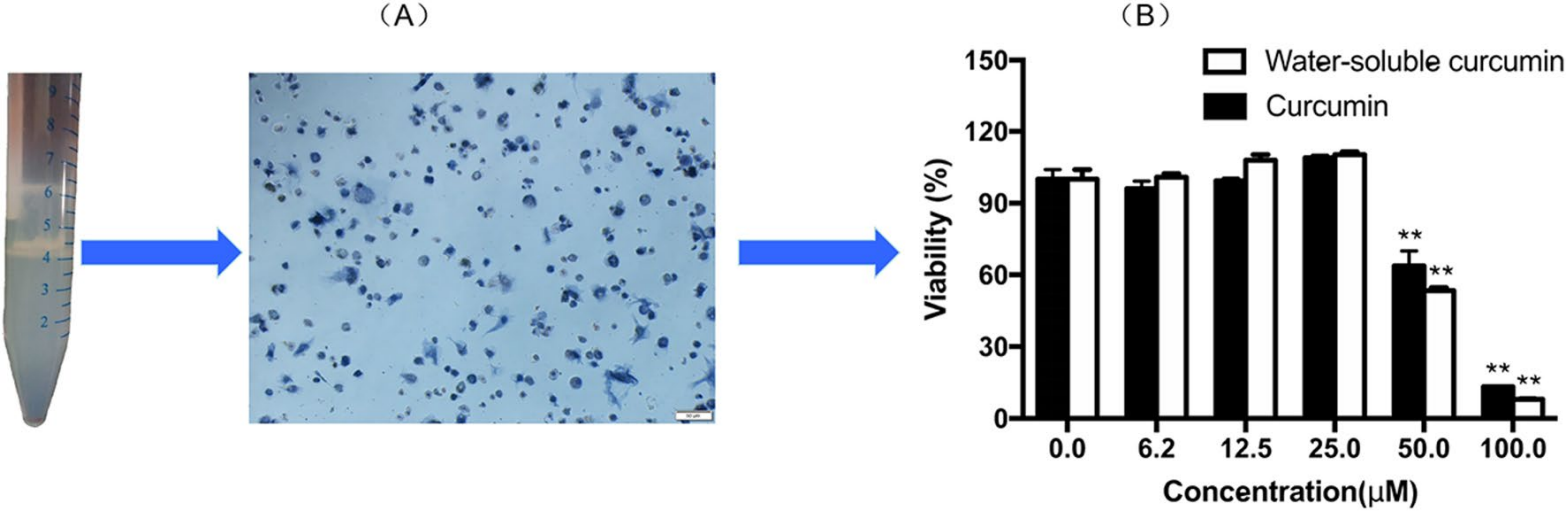
(**A**) 3β-HSD staining of purified rat Leydig cells (located in 4 mL scale). The positive cells were stained in dark blue color. (**B**) The viability of Leydig cells after curcumin or β-cyclodextrin–curcumin treatment.

The 3β-HSD and trypan blue staining showed that Leydig cells were successfully isolated from testes (Fig. 1), with an average of 96.23% viability and an approximate purity of 90%. MTT results showed that 50 μM and 100 μM curcumin and CDC significantly reduced the cell viability compared to untreated control. However, there were no apparent differences at lower concentrations, with ≤ 25 μM CDC or curcumin (Fig. 1). Therefore, the subsequent experiments were performed in concentrations ranging from 0.1 to 25 μM.

### Testosterone production

**Figure 2:**
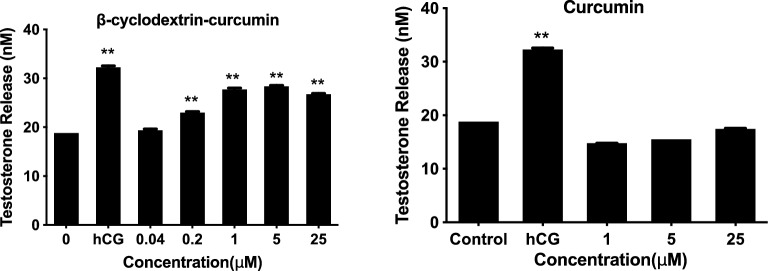
Effects of β-cyclodextrin–curcumin and curcumin on testosterone secretion in Leydig cells. **P < 0.01 respect to Control.

Exposure to 1 IU/mL hCG resulted in a significant increase in levels of testosterone production in rat Leydig cells. Similarly, the CDC played an excellent role in increasing testosterone secretion ranging from 0.2 to 25 μM (Fig. 2), and 5 μM CDC showed the highest effect on testosterone secretion. Besides, curcumin had a somewhat inhibitory impact on steroidogenesis, but there was no significant difference between control and regular curcumin treatment (1–25 μM).

### Changes in mRNA expression levels of transcription factor and steroidogenic enzymes

**Figure 3:**
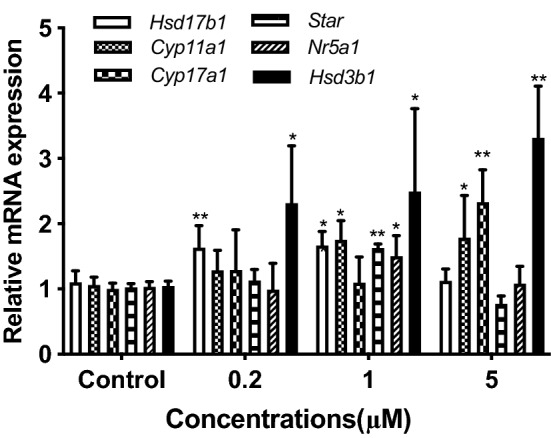
Effects of β-cyclodextrin–curcumin on the mRNA expression levels of *Nr5a1* and steroidogenic enzymes in Leydig cells. *P < 0.05, **P < 0.01 respect to Control.

The above results suggested that the CDC might regulate the expression levels of steroidogenic enzyme genes to promote testosterone secretion in Leydig cells. Therefore, the mRNA expression levels of *Nr5a1* and steroidogenic enzymes (*Star, Cyp11a1, Cyp17a1, Hsd3b1, and Hsd17b1*) were analyzed by RT-qPCR. As shown in Fig. 3, cells exposed to hCG and CDC (1 μM or 5 μM) demonstrated significantly higher expression levels of *Nr5a1* and steroidogenic enzymes compared to controls (*P* < 0.05 or *P* < 0.01). Therefore, the results suggested that the CDC could promote testosterone synthesis by regulating the transcription of the *Nr5a1* gene and activating the expression of steroidogenic enzyme genes.

### Changes in transcription factors and steroidogenic proteins

**Figure 4:**
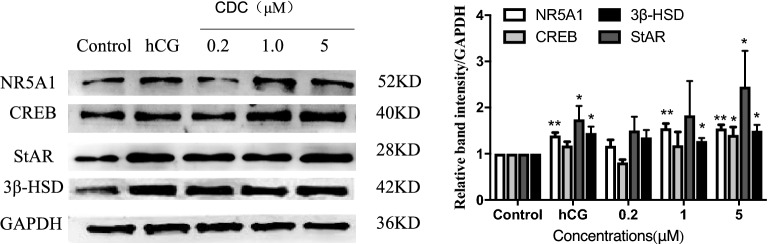
Effect of β-cyclodextrin–curcumin on the protein expression of NR5A1, CREB, StAR, and 3β-HSD in Leydig cells. *P < 0.05, **P < 0.01 respect to Control. *CDC* β-CD-cucurmin.

We next tested if the CDC could affect transcription factors and steroidogenic proteins. Figure 4 and Supplementary Figures showed that hCG and CDC (1 μM or 5 μM) increased the protein expression of NR5A1, CREB, 3β-HSD, and StAR compared to controls (*P* < 0.05 or *P* < 0.01).

**Figure 5:**
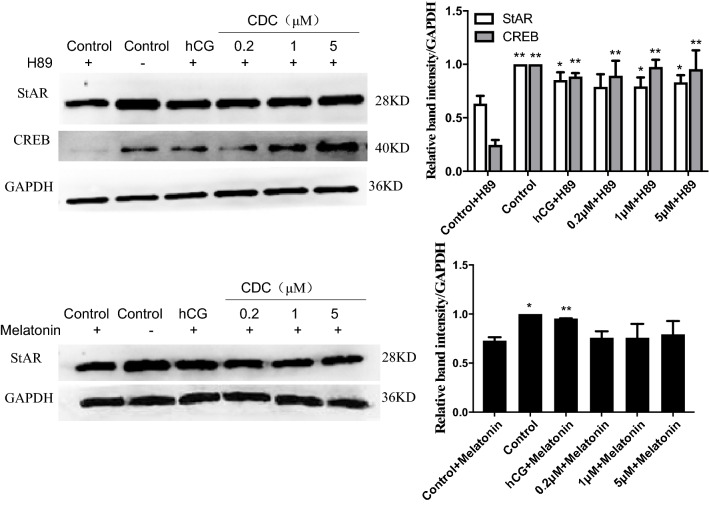
Effect of β-cyclodextrin–curcumin on the protein expression of StAR and CREB following the addition of H89 or melatonin in Leydig cells. *P < 0.05, **P < 0.01 respect to control + inhibitor. *CDC* β-CD-cucurmin.

H89 is a selective and strong inhibitor of cAMP-activated protein kinase A (PKA) via competitive antagonists of the ATP site on the PKA catalytic subunit^29^. Melatonin, an indolamine neurohormone, can decrease testicular androgen synthesis and the size of testes through melatonin subtype 1a (mel1a) receptors and the local corticotrophin-releasing hormone system^30^. These data indicate that 1-h pretreatment with H89 resulted in a significant diminution in the protein expression of CREB and StAR compared with their expressions in control cells (*P* < 0.01) (Fig. 5) and Supplementary Figures. Treatment with hCG or 0.2–5 μM CDC restored the H89-inhibited CREB and StAR levels (*P* < 0.05 or *P* < 0.01). Similarly, the addition of melatonin to Leydig cells prevented the StAR level (*P* < 0.05) compared to control. HCG could recover melatonin-inhibited StAR protein expression (*P* < 0.01), but the CDC had no significant effect on it (*P* > 0.05).

### XenoScreen YES/YAS assay

**Figure 6:**
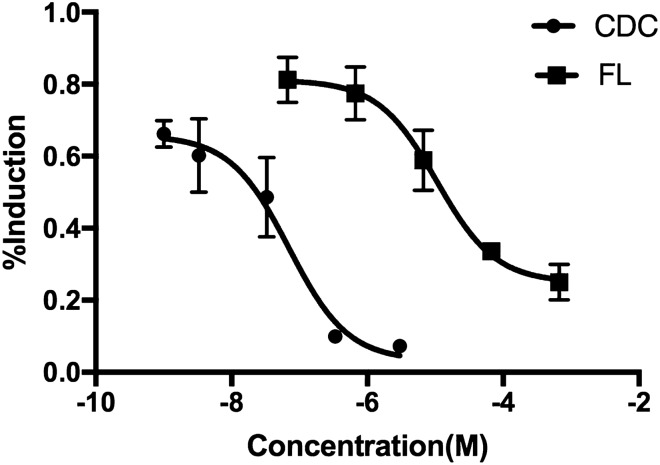
Androgenic antagonistic activity of β-cyclodextrin–curcumin. *CDC* β-CD-cucurmin, *FL* flutamide.

The CDC interacting with hER or hAR was assessed through XenoScreen XL YES/YAS assay. The results revealed no apparent cytotoxicity under the testing concentrations of 100 μM. As shown in Fig. 6, CDC showed androgenic antagonistic activity as flutamide. The IC50 values of flutamide and CDC were 1.05 × 10^–5^ M and 7.00 × 10^–8^ M, respectively. However, no estrogenic agonistic, androgenic agonistic, and estrogenic antagonistic potential of CDC was observed below 100 μM (Table 2).

**Table 2:** EC50 or IC50 values.

Group	Sample	EC50/IC50(M)	95%confidence interval (M)	R2
ER agonists	E2	2.63 × 10^–10^	1.60 × 10^–10^–4.29 × 10^–10^	0.9894
	CDC	–	–	–
AR agonists	DHT	2.49 × 10^–9^	1.85 × 10^–9^–3.40 × 10^–9^	0.9541
	CDC	–	–	–
ER antagonists	4-HT	2.65 × 10^–7^	1.35 × 10^–7^–5.20 × 10^–7^	0.9918
	CDC	–	–	–
AR antagonists	FL	1.05 × 10^–5^	3.21 × 10^–6^–3.88 × 10^–5^	0.9311
	CDC	7.00 × 10^–8^	2.01 × 10^–8^–2.44 × 10^–7^	0.9167

*ER* estrogenic receptor, *AR* androgenic receptor, *E2* 17β-estradiol, *4-HT* 4-hydroxytamoxifen, *DHT* 5α-dihydrotestosterone, *FL* flutamide.

### Effect of CDC on sexual behavior, hormone levels, and organ coefficient

**Figure 7:**
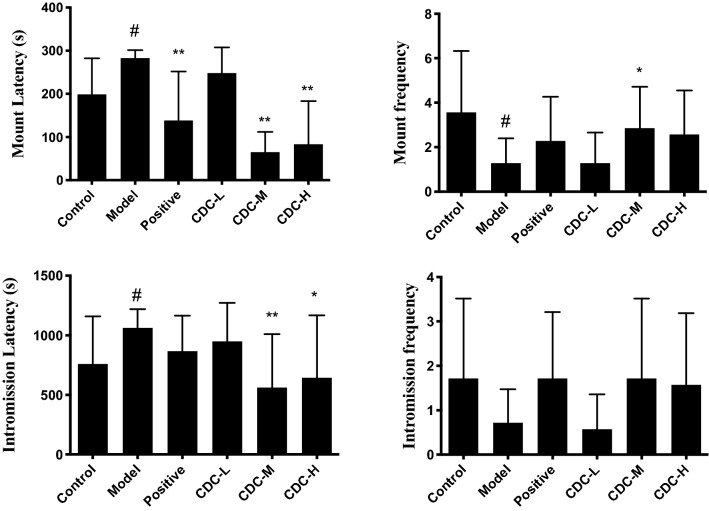
Effect of β-cyclodextrin–curcumin on the mount latency, mount frequency, intromission latency, and intromission frequency of hydrocortisone treated male mice. *P < 0.05, **P < 0.01 respect to Model; ^#^*P* < 0.05, ^##^*P* < 0.01 respect to Control. *Control* normal control group, *Model* hydrocortisone control group, *Positive* Jinkui Shenqi Pill group, *CDC-L* low dose of β-CD-curcumin group, *CDC-M* medium dose of β-CD-curcumin group, *CDC-H* high dose of β-CD-curcumin group.

**Figure 8:**
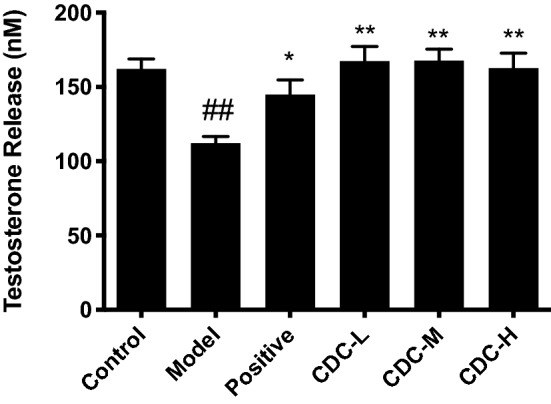
Effect of β-cyclodextrin–curcumin on the testosterone concentration of hydrocortisone treated male mice. *P < 0.05, **P < 0.01 respect to Model; ^#^*P* < 0.05, ^##^*P* < 0.01 respect to Control. *Control* normal control group, *Model* hydrocortisone control group, *Positive* Jinkui Shenqi Pill group, *CDC-L* low dose of β-CD-curcumin group, *CDC*-*M* medium dose of β-CD-curcumin group, *CDC-H* high dose of β-CD-curcumin group.

Compared with the blank control group, hydrocortisone treatment decreased the copulatory performance of sexually experienced male mice: mount and intromission latencies were significantly increased (*P* < 0.05), and mount and intromission frequency were significantly reduced (*P* < 0.05) (Fig. 7). Compared with the model animal group, CDC (in medium and high doses) and JKSQP groups reduced the mount and intromission latencies while increased mount and intromission frequency significantly (*P* < 0.05 or *P* < 0.01). The hydrocortisone administration exhibited a decrease in serum testosterone concentration (*P* < 0.01), while after CDC or JKSQP resulted in a significant increase (*P* < 0.01) (Fig. 8).

**Figure 9:**
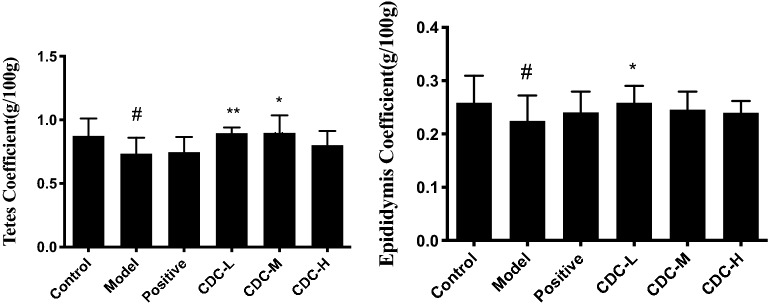
Effect of β-cyclodextrin–curcumin on organ coefficient of hydrocortisone treated male mice. *P < 0.05, **P < 0.01 respect to Model; ^#^*P* < 0.05, ^##^*P* < 0.01 respect to Control. *Control* normal control group, *Model* hydrocortisone control group, *Positive* Jinkui Shenqi Pill group, *CDC-L* low dose of β-CD-curcumin group, *CDC-M* medium dose of β-CD-curcumin group, *CDC-H* high dose of β-CD-curcumin group.

The weight of testes and epididymis showed a significant decrease in the hydrocortisone-treated mice compared to the control group (*P* < 0.05) (Fig. 9). A significantly increased weight of testes and epididymis was observed in CDC groups compared with hydrocortisone-treated mice (*P* < 0.05 or *P* < 0.01).

### Effect of CDC on histological changes in testicular tissue

**Figure 10:**
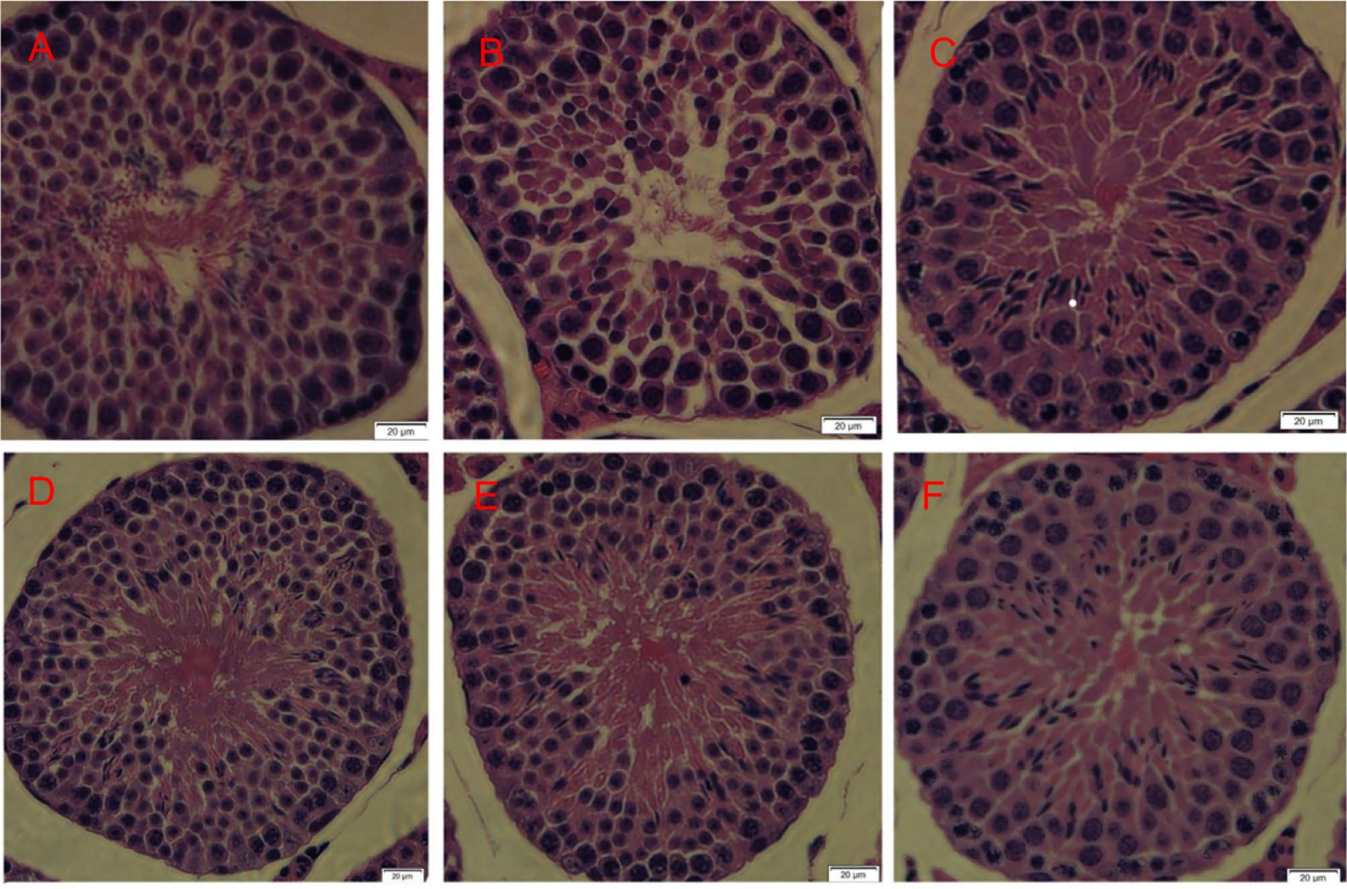
HE staining of testicular tissue in mice (×400). (**A**) Normal control group, (**B**) hydrocortisone control group, (**C**) Jinkui Shenqi Pill group, (**D**) low dose of β-CD-curcumin group, (**E**) medium dose of β-CD-curcumin group, (**F**) high dose of β-CD-curcumin group.

Histological changes in mouse testis are shown in Fig. 10. The control mice exhibited a normal process of spermatogenesis with a regular arrangement of the spermatogenic epithelium in the seminiferous tubules. The model group showed various testicular changes, including the loss, and disorder of the spermatogenic cells, the decrease of spermatogenesis, as well as sloughing in the cytoplasm of Sertoli cells. However, the tubules in the CDC (in medium and high doses) and JKSQP groups restored their regular shape with comparatively well-organized spermatogenic layers and a moderate amount of sperm. These groups also showed histologic characteristics similar to those of the control group.

## Discussion

Curcumin administration is hampered by its low solubility and stability in water. The water-soluble curcumin complex with β-CD is used in pharmaceutical applications and as a natural food colorant^31^. Although the hydrophilic curcumin from different companies varied in curcumin content, we found they had a similar effect on rat testicular Leydig cells. However, the regular curcumin was not as sensitive as CDC, and it had no significant effect on Leydig cells. These results were similar to the reported that curcumin had no impact on the basal steroidogenesis in MA-10 cells, but it could inhibit the steroidogenesis in primary mouse Leydig cells^32^. Meanwhile, the 5 μM CDC showed higher testosterone levels, and the subsequent experiments were performed in concentrations ranging from 0.1 to 5 μM.

To explore the aphrodisiac effect of CDC in vivo, we used hydrocortisone to establish the Kidney–Yang deficiency model. The metabolic pathways and endocrine and reproductive systems were disturbed by hydrocortisone^33^. Compared with the control group, the subcutaneous hydrocortisone injection in mice could lead to Kidney–Yang deficiency, evidenced by prolonged sexual arousal, reducing the number of sexual encounters, the reproductive organs’ indexes, and testosterone concentration, as well as pathological damage to the testicular tissue structure. After the administration of the CDC, mice mating ability with impotence, reproductive organ weights, testosterone level, and pathological damage was significantly improved. These indicated that the CDC had an aphrodisiac effect on a hydrocortisone-induced mice model of Kidney–Yang deficiency.

Testosterone is synthesized from cholesterol in a multi-step enzymatic response to pituitary hormones. Our data showed that CDC stimulated testosterone production via cAMP/PKA signaling pathways, upregulating transcription factors (NR5A1 and CREB), and steroidogenic enzymes (StAR, CYP11A1, CYP17A1, and 3β/17β-HSD). Steroidogenesis is mediated in cAMP/PKA-dependent and -independent signaling pathways^34^. H89 could down-regulate StAR expression via selective inhibiting PKA activity, while melatonin has a significant protective and regulatory role in Leydig cells. The effects of melatonin on the levels of reproductive hormones depend on physiological conditions and species of animals^35^. Melatonin inhibits StAR, GATA-4/SF-1, or other protein expressions through specific binding sites by blocking StAR protein expression without altering the activity of the P450 enzyme^36–38^. Meanwhile, melatonin promotes male reproductive performance and increases testosterone synthesis in mammalian Leydig cells^39^. The previous study reported that the effect of melatonin on oxytocin and vasopressin release could not entirely be blocked by H89^40^, and it regulated CRE-dependent gene transcription underlying osteoblast proliferation via activating Src and PKA in parallel^41^. Our results demonstrated that the CDC could significantly upregulate H89-suppressed StAR and CREB expression and could not reverse melatonin-suppressed StAR expression. This result was strengthened by previous evidence that determined H89 abolished the effect of curcumin treatment to increase phosphorylation of LKB-1 and CREB^42^. Thus, the CDC seemed to stimulate steroidogenesis via the cAMP/PKA signaling pathway, not affected by the cAMP synthesis inhibitor.

The present study further showed that the CDC had androgenic antagonistic activity on transgenic yeast without estrogenic agonistic, androgenic agonistic, and estrogenic antagonistic potential. This means that the CDC might exert two-way regulation in the biological effects of hormones in the body. It is an androgen receptor antagonist with potential as an anti-prostate cancer agent^43^ and could also stimulate androgen production through the steroidogenic pathway.

## Conclusion

**Figure 11:**
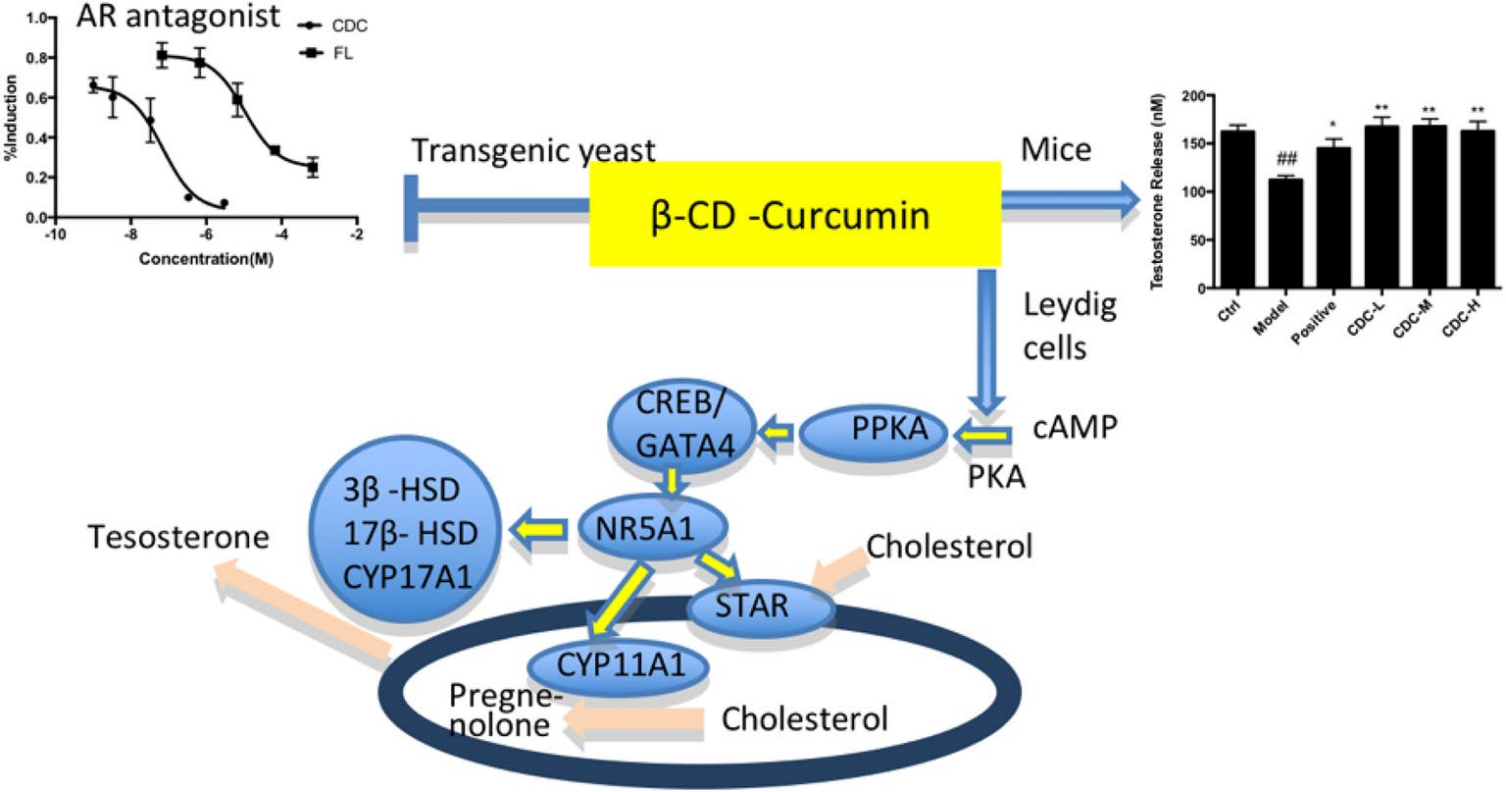
The aphrodisiac potential of β-cyclodextrin–curcumin via stimulating cAMP-PKA pathway in testicular Leydig cells.

The in vitro study showed that the CDC stimulated testosterone production via the cAMP/PKA signaling pathway. In vivo, CDC had an aphrodisiac effect on a hydrocortisone-induced mouse model of Kidney–Yang deficiency, evidenced by significantly improving the sexual abilities of mice with impotence, reproductive organ weights, testosterone level, and pathological damage. Besides, CDC showed potential androgenic antagonistic activity on transgenic yeast. These results mean that the CDC might exert two-way regulation in the biological effects of hormones in the body (Fig. 11).

## Supplementary Information

Supplementary Figures.

## Data Availability

The data that support the findings of this study are available from the corresponding author upon reasonable request.

## Abbreviations

cAMP: Cyclic adenosine monophosphate
CDC: β-Cyclodextrin–curcumin
CREB: CAMP-response element binding protein
CYP11A1: Cholesterol side-chain cleavage enzyme
CYP17A1: 17-Alpha-hydroxylase/17,20-lyase
3β/17β-HSD (HSD3B1/HSD17B1): 3β-/17β-Hydroxysteroid dehydrogenase type 1
DHT: 5α-Dihydrotestosterone
E2: 17β-Estradiol
FL: Flutamide
hAR: Human androgenic receptor
HE: Hematoxylin and eosin
hER: Human estrogenic receptor
HPLC: High-performance liquid chromatography
4-HT: 4-Hydroxytamoxifen
IF: Intromission frequency
IL: Intromission latency
JKSQP: Jinkui Shenqi Pill
MF: Mount frequency
ML: Mount latency
NR5A1: Steroidogenic factor-1
PKA: Protein kinase A
RT-PCR: Real-time reverse transcription polymerase chain reaction
StAR: Steroidogenic acute regulatory protein
YAS: Yeast androgen screen
YES: Yeast estrogen screen
