# Supplementary material for: The aphrodisiac potential of β-cyclodextrin–curcumin via stimulating cAMP-PKA pathway in testicular Leydig cells
Source: Sci Rep. 2022 Aug 22;12:14263. doi: 10.1038/s41598-022-18065-3 (PMC9395524; doi:10.1038/s41598-022-18065-3)
Supplement: Supplementary file 1 — Supplementary Figures. [file 41598_2022_18065_MOESM1_ESM.pdf]

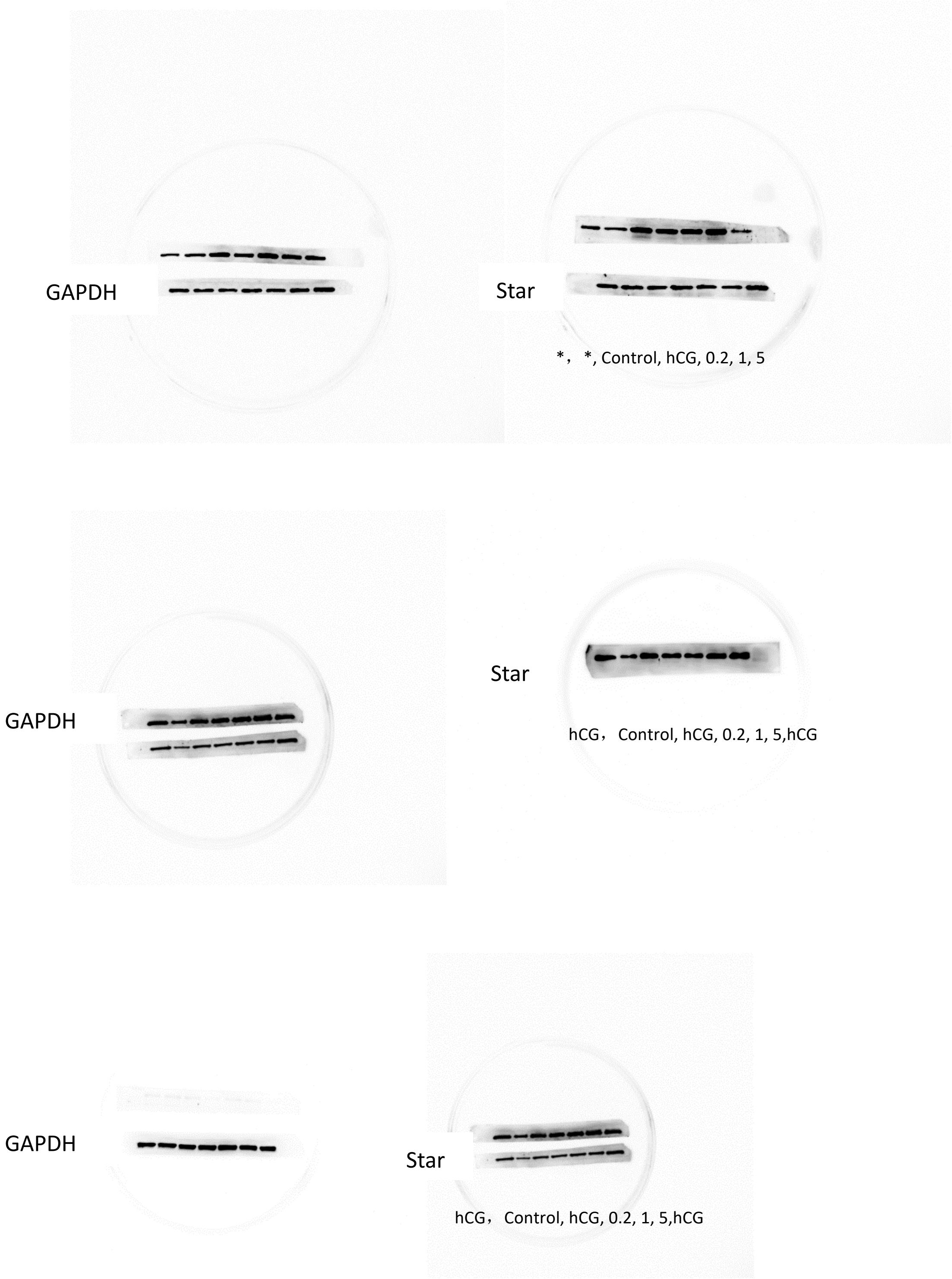

Figure 4-Effect of β-cyclodextrin-curcumin on the protein expression of StAR in Leydig cells.

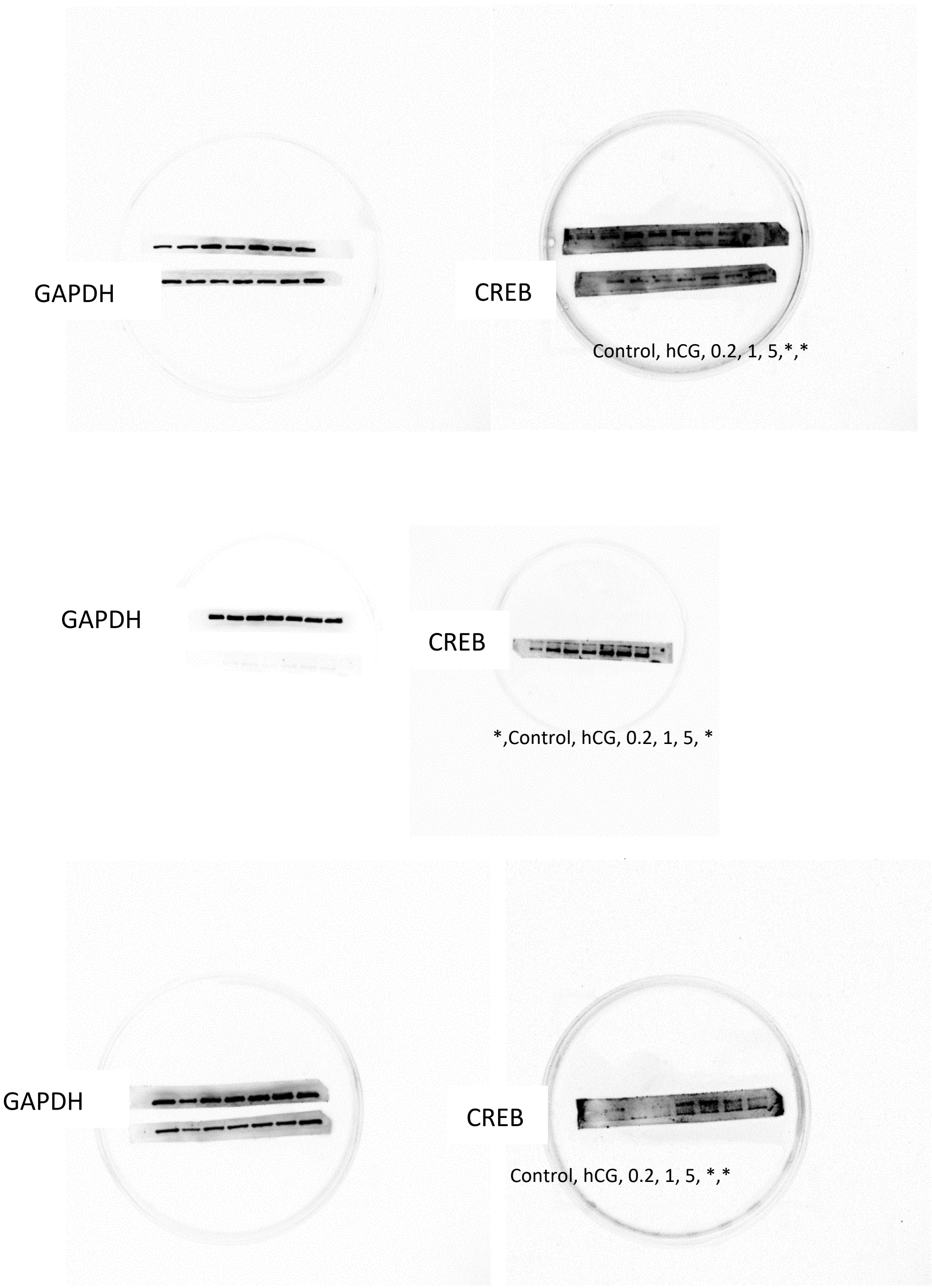

Figure 4-Effect of β-cyclodextrin-curcumin on the protein expression of CREB in Leydig cells.

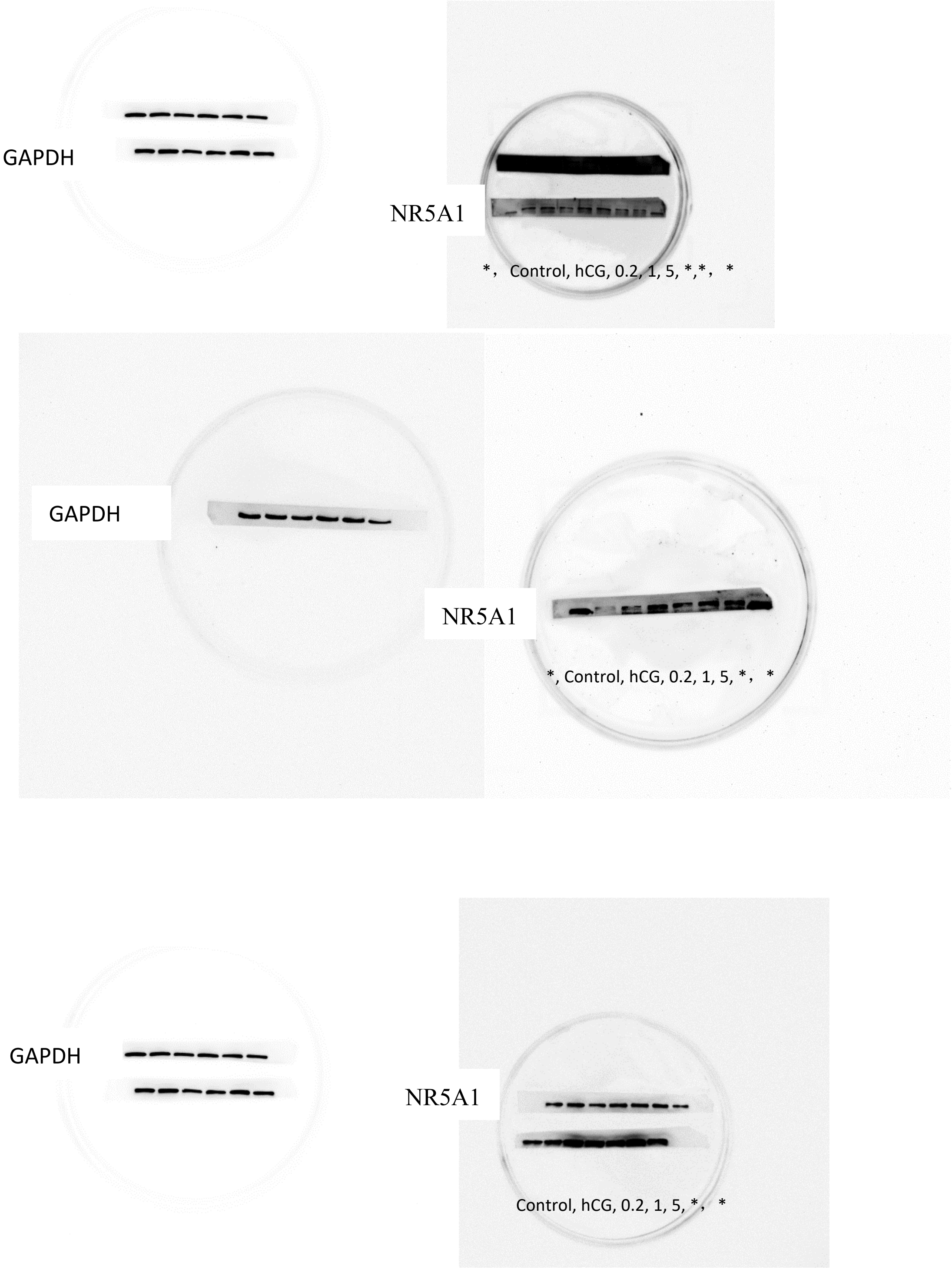

Figure 4-Effect of β-cyclodextrin-curcumin on the protein expression of NR5A1 in Leydig cells.

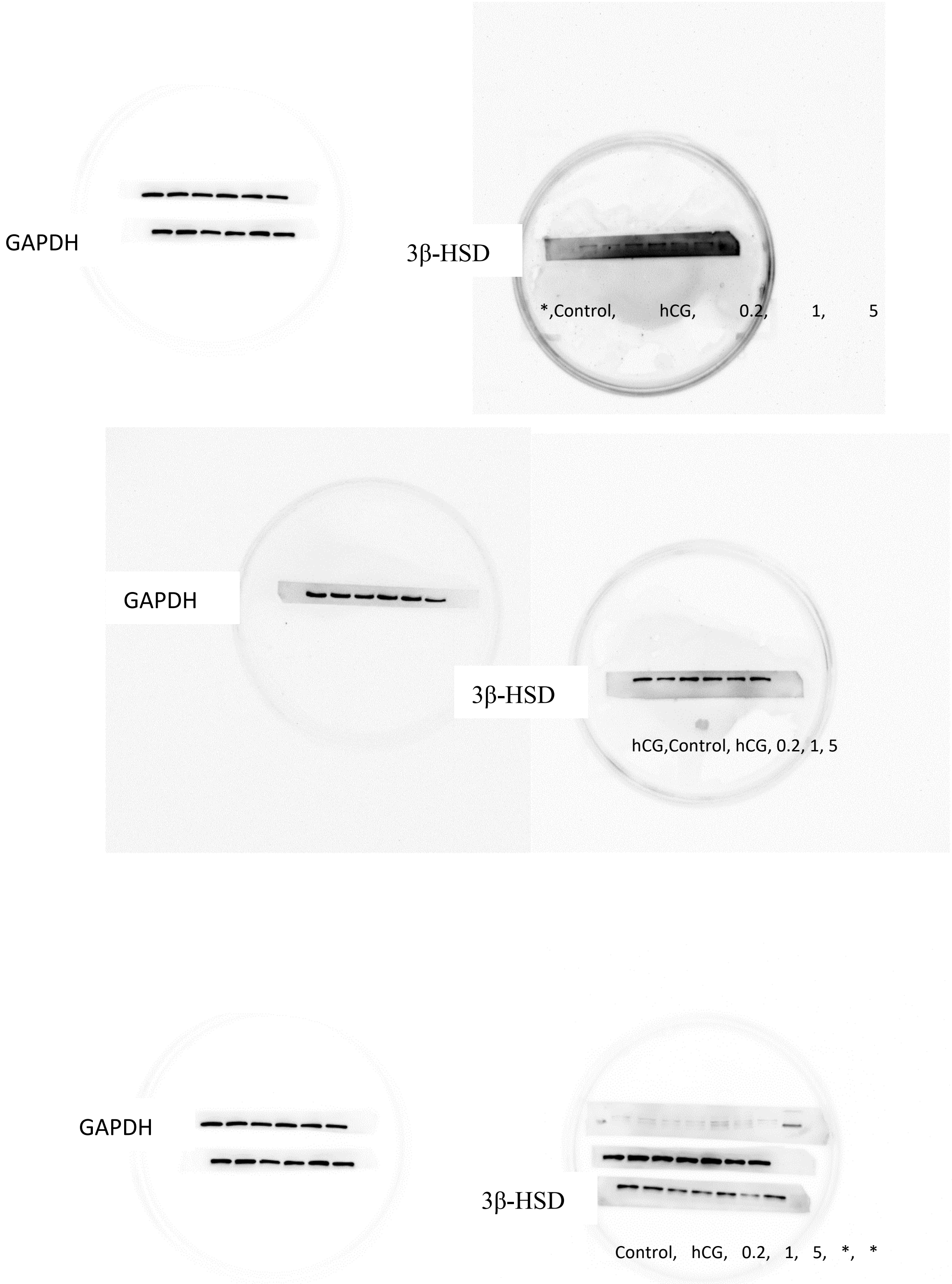

Figure 4-Effect of β-cyclodextrin-curcumin on the protein expression of 3β-HSD in Leydig cells.

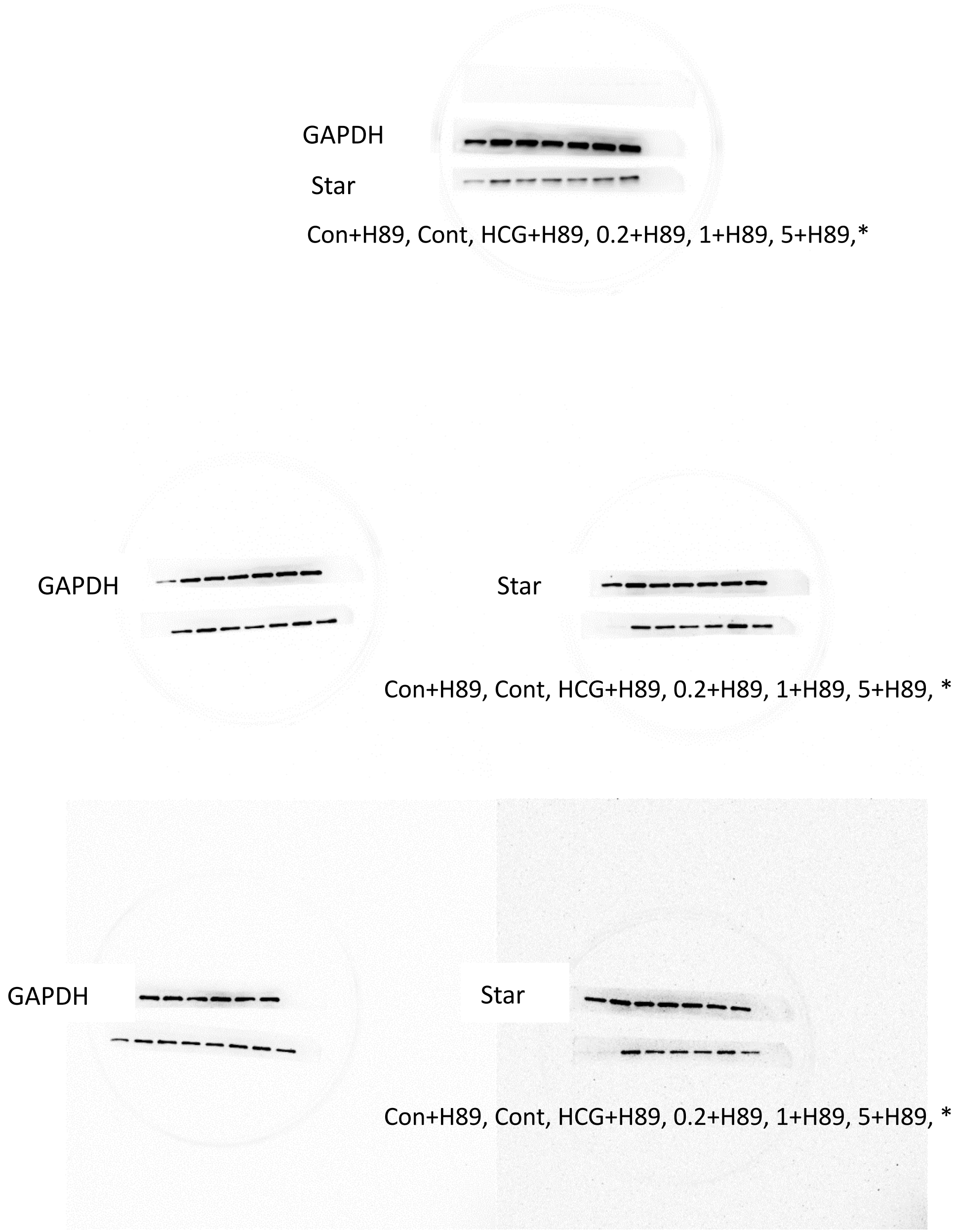

Figure 5-Effect of β-cyclodextrin-curcumin on the protein expression of StAR following the addition of H89 in Leydig cells.

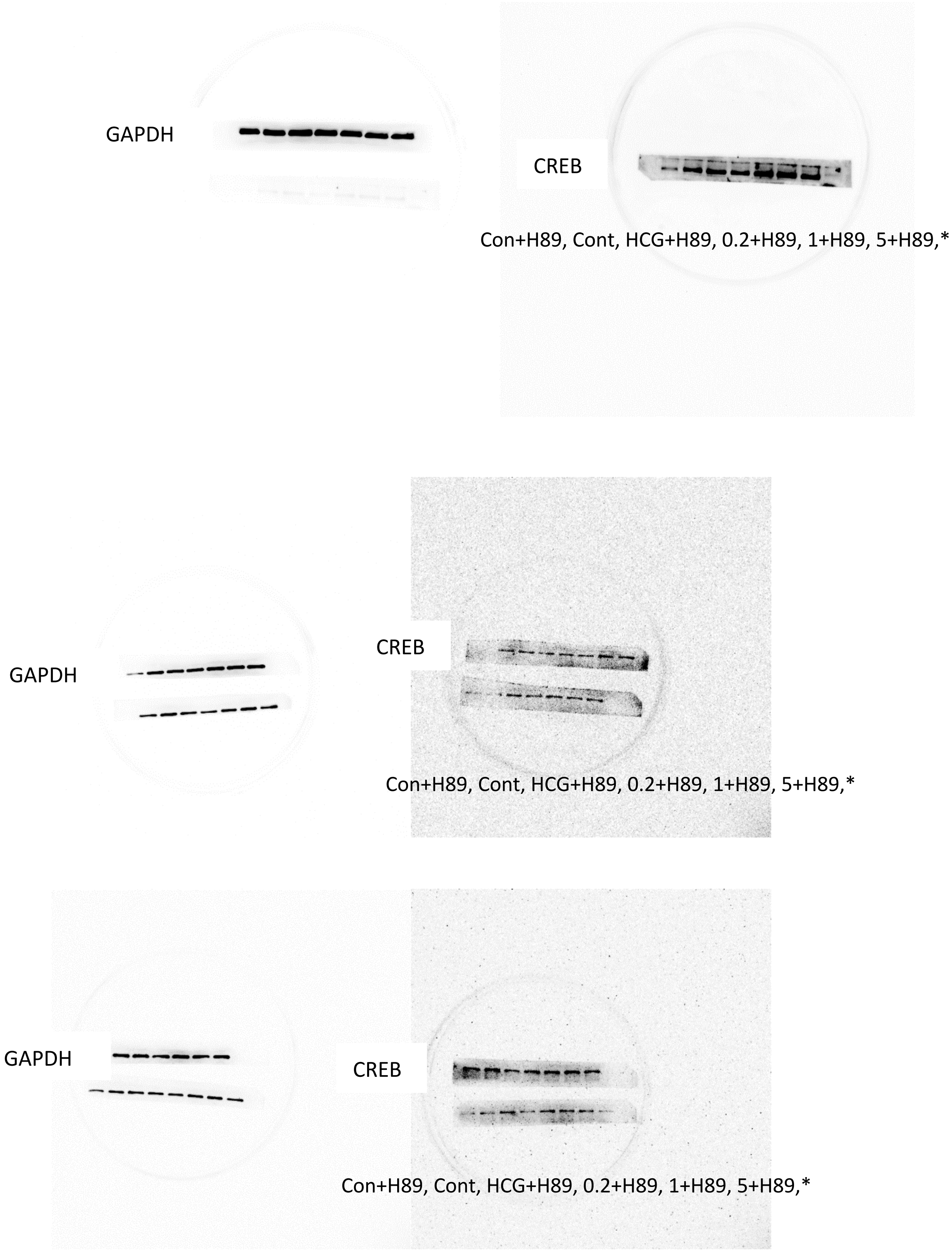

Figure 5-Effect of β-cyclodextrin-curcumin on the protein expression of CREB following the addition of H89 in Leydig cells.

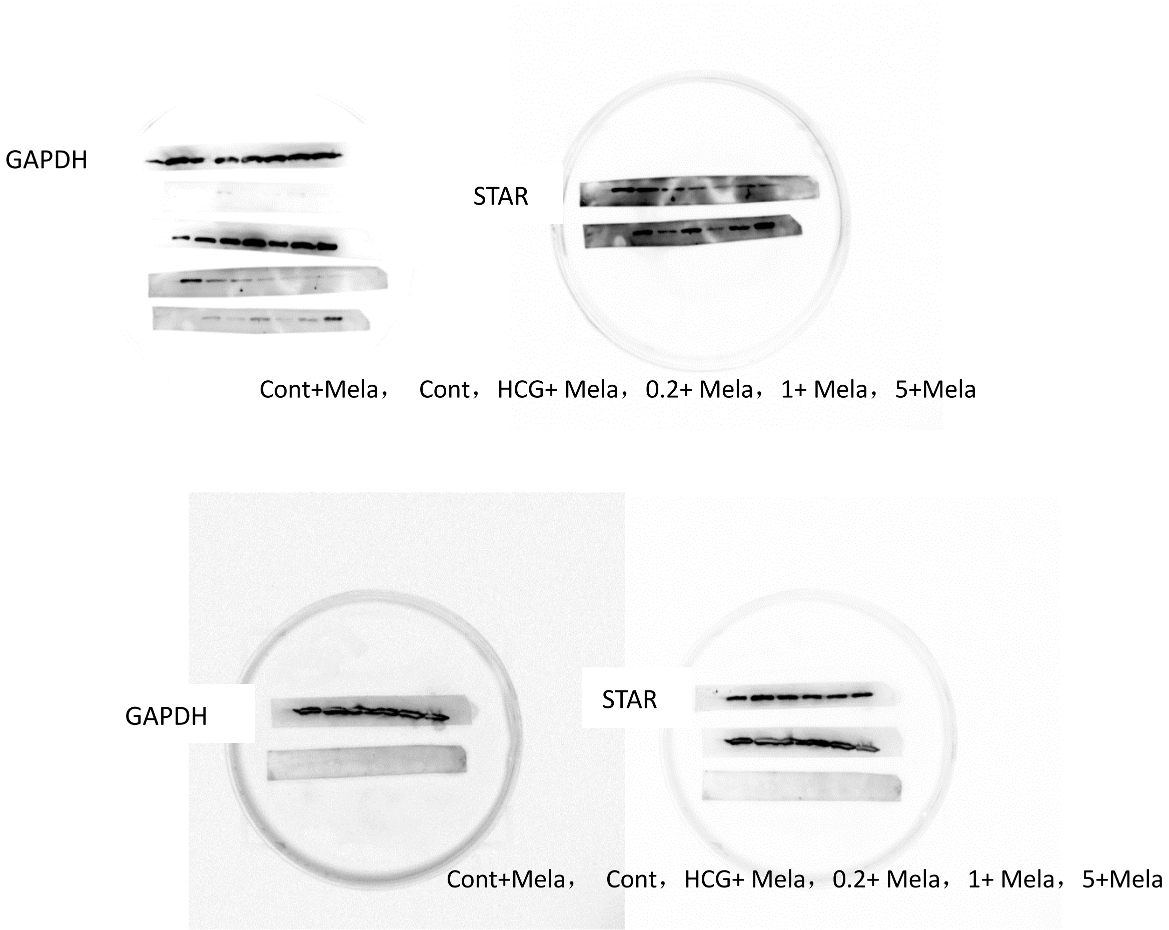

Figure 5-Effect of β-cyclodextrin-curcumin on the protein expression of StAR following the addition of melatonin in Leydig cells.
